# Year-round spawning of three tropical Cypriniformes fishes in Southeast Asia

**DOI:** 10.1038/s41598-023-36065-9

**Published:** 2023-06-02

**Authors:** Rafhiah Kahar, Norhayati Ahmad, Takaomi Arai

**Affiliations:** 1 Institute for Biodiversity and Environmental Research, Universiti Brunei Darussalam, Jalan Tungku Link, Gadong, BE1410 Brunei Darussalam; 2 Environmental and Life Sciences Programme, Faculty of Science, Universiti Brunei Darussalam, Jalan Tungku Link, Gadong, BE1410 Brunei Darussalam

**Keywords:** Freshwater ecology, Freshwater ecology

## Abstract

Present knowledge on spawning seasonality of freshwater fishes in tropical Asia and their relationship with environmental factors remains limited. Three Southeast Asian Cypriniformes fishes, *Lobocheilos ovalis*, *Rasbora argyrotaenia* and *Tor Tambra*, found in rainforest streams in Brunei Darussalam were studied on a monthly basis for a period of 2 years. To assess spawning characteristics, seasonality, gonadosomatic index and reproductive phases were examined from 621 *L. ovalis*, 507 *R. argyrotaenia* and 138 *T. tambra*. This study also examined environmental factors such as rainfall, air temperature, photoperiod and lunar illumination that may influence the timing of spawning of these species. We found that *L. ovalis*, *R. argyrotaenia* and *T. tambra* were reproductively active throughout the year but did not find that spawning in these species were associated with any of the investigated environmental factors. Our study showed that the non-seasonal reproductive ecology found in the tropical cypriniform species is distinctly different from that of temperate cypriniforms, which are known to follow spawning seasonality, suggesting an evolutionary adaptation to ensure their survival in an unstable environment. The reproductive strategy and ecological responses found in the tropical cypriniforms might be shifted in response to climate change scenarios in the future.

## Introduction

Present knowledge on spawning seasonality in freshwater fishes is largely based on temperate species in North America and Europe as well as tropical species in South America. Considerably less information is available on the spawning seasonality of freshwater fishes in tropical Asia, particularly in the Southeast Asian region. In temperate latitudes, fishes often show some degree of seasonality in reproduction and limit their annual spawning within a short but optimal period^1–3^. Spawning in these freshwater fishes often occurs in months with warmer temperatures and longer day lengths^4–7^. In the warm and wet tropics, freshwater fishes commonly exhibit continuous reproduction or reduced seasonality^2,8,9^. Protracted spawning in tropical species often coincides with the wet season^10–14^.

Cypriniformes is a diverse order of freshwater fishes^15^ that characterizes the freshwater fish fauna of tropical Asia^16,17^. In this region, spawning in some cypriniform species extend throughout the year while also displaying peak spawning activities as described in *Barbus nigrofasciatus*, *Barbus dorsalis*, *Barbus titteya*^9^, *Neolissochilus soroide*s^18^, and *Rasbora tawarensis*^19^. Conversely, seasonal spawning has also been reported in other tropical Asian cypriniform fishes such as in *Tor tambroides*^20^, *Barbus bimaculatus*, *Barbus cumingii*, *Puntius vittatus*^9^ and *Barbus lacerta*^21^. These studies have reported that peaks as well as seasonality in spawning coincide with periods of high rainfall, further confirming rainfall as an important environmental cue for reproduction in tropical freshwater fishes. However, few studies on freshwater fishes in tropical Asia have examined the association of spawning with other environmental factors such as temperature, photoperiod and lunar illumination. While air temperature does not show major seasonal variations in the tropical regions, it has been shown to influence reproductive activities in Neotropical and South Asian freshwater fishes^12,22,23^. Longer photoperiod has been reported to trigger spawning migration of Neotropical riverine fishes^24–26^.

The present study examines the reproductive ecology of Southeast Asian Cypriniformes fishes *Lobocheilos ovalis*, *Rasbora argyrotaenia* and *Tor tambra* belonging to the Cyprinidae family^17,27^. However, in a recent phylogenetic study the *Rasbora* genus has been reclassified in the Danionidae family^28^. Southeast Asia is one of the world’s biodiversity hotspots and represents a region of distinct conservation importance^29^. It is estimated that 70 of the 205 genera of freshwater fishes belonging to the Cyprinidae family are endemic to this region^16^. Unprecedented rates of habitat degradation and over exploitation have been recognized as major threats affecting the natural populations of freshwater fishes in Southeast Asia^30^. Although categorized as ‘Least Concern’ in the IUCN red list of threatened species (IUCN red list)^31^, the limited geographical range of *L. ovalis* in Northwest Borneo^32^ makes this species susceptible to extinction in the face of habitat degradation and climate change^33^. Similarly, *R. argyrotaenia* is a species of ‘Least Concern’^34^ but has recently been recognized as a potential aquaculture species^35–37^. While, *T. tambra* is categorized as ‘Data Deficient’ according to the IUCN red list, the wild population is known to be declining due to excessive exploitation^38^.

There is very scarce information available on the reproductive ecology of *L. ovalis*, *R. argyrotaenia* and *T. tambra*. Thus, our aim is to investigate the spawning seasonality in these species using monthly captured specimens. We examined spawning seasonality by evaluating the gonadosomatic index (GSI) and reproductive phases of the gonads. We also examined environmental factors such as rainfall, air temperature, photoperiod and lunar illumination influencing the timing of spawning in these species. Our findings contributed to further understanding of the life history strategies of tropical cypriniform fishes which inhabit flashy and unstable headwater streams. These findings suggest the utility of life history knowledge for interpreting ecological responses in freshwater fishes in the context of habitat and climate changes.

## Results

### Sex composition, total length (TL) and gonadosomatic index (GSI)

A total of 724 *L. ovalis*, 609 *R. argyrotaenia* and 145 *T. tambra* were collected in this study. There was a total of 353 female and 268 male *L. ovalis*, while the sexes of 103 specimens could not be determined. For *R. argyrotaenia* a total of 335 females and 172 males were found, while sexes of 102 specimens could not be determined. A total 120 female and 18 male of *T. tambra* were found, while the sexes of 7 specimens could not be determined. Gonads that could not be determined were either undeveloped or damaged during processing. Specimens of 621 *L. ovalis*, 507 *R. argyrotaenia* and 138 *T. tambra* were used for further analysis (Table 1). In the three species, overall sex compositions were female dominant (Chi-squared test, P < 0.05).

**Table 1:** Sample size, total length (TL) and gonadosomatic index (GSI) in *L. ovalis*, *R. argyrotaenia* and *T. tambra* collected from the Temburong Basin in September 2017 to August 2019. Values of TL and GSI are expressed as range. na: not available.

Reproductive phase	Sample size		TL (mm)		GSI	
	Female	Male	Female	Male	Female	Male
*L. ovalis*	353	268	145–269	154–269	0.02–18.82	0.05–11.04
Immature	4	1	155–180	169	0.21–0.99	0.99
Developing	20	5	172–238	165–208	0.46–1.71	0.54–4.47
Spawning capable	14	71	172–260	158–257	1.77–14.3	0.77–10.75
Actively spawning	102	124	200–269	154–269	1.50–18.82	0.24–11.04
Regressing	127	40	203–257	157–204	0.02–6.01	0.11–5.12
Regenerating	59	15	145–250	16–206	0.16–3.3	0.05–2.25
*R. argyrotaenia*	335	172	93–162	95–134	0.01–17.66	0.07–16.99
Immature	21	1	93–123	95–123	0.01–1.64	0.07
Developing	19	2	105–155	97–100	0.04–1.71	2.39–4.07
Spawning capable	25	58	95–153	88–144	0.77–12.87	0.85–12.12
Actively spawning	169	94	95–162	87–123	0.18–17.66	0.16–16.99
Regressing	64	10	95–156	95–119	0.10–8.99	0.15–8.97
Regenerating	38	5	95–43	101–134	0.03–5.73	0.12–0.28
*T. tambra*	120	18	184–487	200–345	0.05–6.78	0.12–2.03
Immature	60	0	184–415	na	0.09–1.39	na
Developing	13	2	275–410	301–309	0.19–1.11	0.27–1.11
Spawning capable	3	10	277–487	244–345	1.87–7.39	0.26–1.61
Actively spawning	4	2	281–460	277–287	0.18–6.78	1.52–2.03
Regressing	5	1	220–310	282	0.26–0.47	0.75
Regenerating	33	2	200–402	200–246	0.05–0.53	0.12–0.28

All species showed sexual dimorphism where the females were larger than males (LME analyses; P < 0.05; Table 1). Female and male GSI were significantly different in all species (LME analyses; P < 0.05; 3.73 ± 0.25 and 3.40 ± 1.30 in female and male *L. ovalis* respectively, 3.97 ± 0.20 and 5.30 ± 0.28 in female and male *R. argyrotaenia* respectively, 0.45 ± 0.09 and 0.84 ± 0.12 in female and male *T. tambra* respectively). All species displayed the highest GSI values during the spawning capable and actively spawning phases (LME analyses, P < 0.05; Table 1).

### Monthly GSI and maturity level frequencies

**Figure 1:**
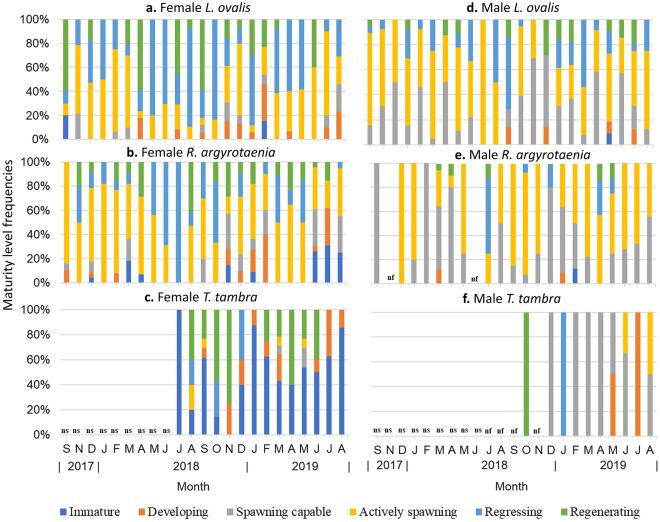
Monthly fluctuations in reproductive phases in: (**a**) Female *L. ovalis*, (**b**) female *R. argyrotaenia*, (**c**) female *T. tambra*, (**d**) male *L. ovalis*, (**e**) male *R. argyrotaenia*, (**f**) male *T. tambra* from September 2017 to August 2019. ns: no study was conducted in the respective month. nf: no fish specimens were collected but study was conducted.

Female GSIs ranged from 0.02 to 18.82 in *L. ovalis*, 0.01 to 17.66 *R. argyrotaenia* and 0.05–6.78 *T. tambra*. Male GSI ranged from 0.05 to 11.04 in *L. ovalis*, 0.07–16.99 in *R. argyrotaenia* and 0.12–2.03 in *T. tambra* (Table 1). The GSI values in female and male *L. ovalis* and *R. argyrotaenia* were highly variable between months (Supplementary Fig. S1). Female and male *L. ovalis* and *R. argyrotaenia* with advanced gonad development i.e., those in the spawning capable and actively spawning phases were found in every month and showed variable frequencies between months (Fig. 1). Similarly, the GSI values in male *T. tambra* were highly variable between months, with the spawning capable and actively spawning phases found nearly in every month. On the other hand, the monthly female GSI values in *T. tambra* were generally low except in March and May 2019, where values above 4 were recorded (Supplementary Fig. S1). Spawning capable and actively spawning female *T. tambra* were only found between August to September 2018 and March and May 2019 (Fig. 1). However, the presence of regressing and developing female *T. tambra* in various months provided indications of spawning in previous and subsequent months (Fig. 1). These findings suggest that *L. ovalis*, *R. argyrotaenia* and *T. tambra* were reproductively active throughout the year.

### Environmental factors

**Figure 2:**
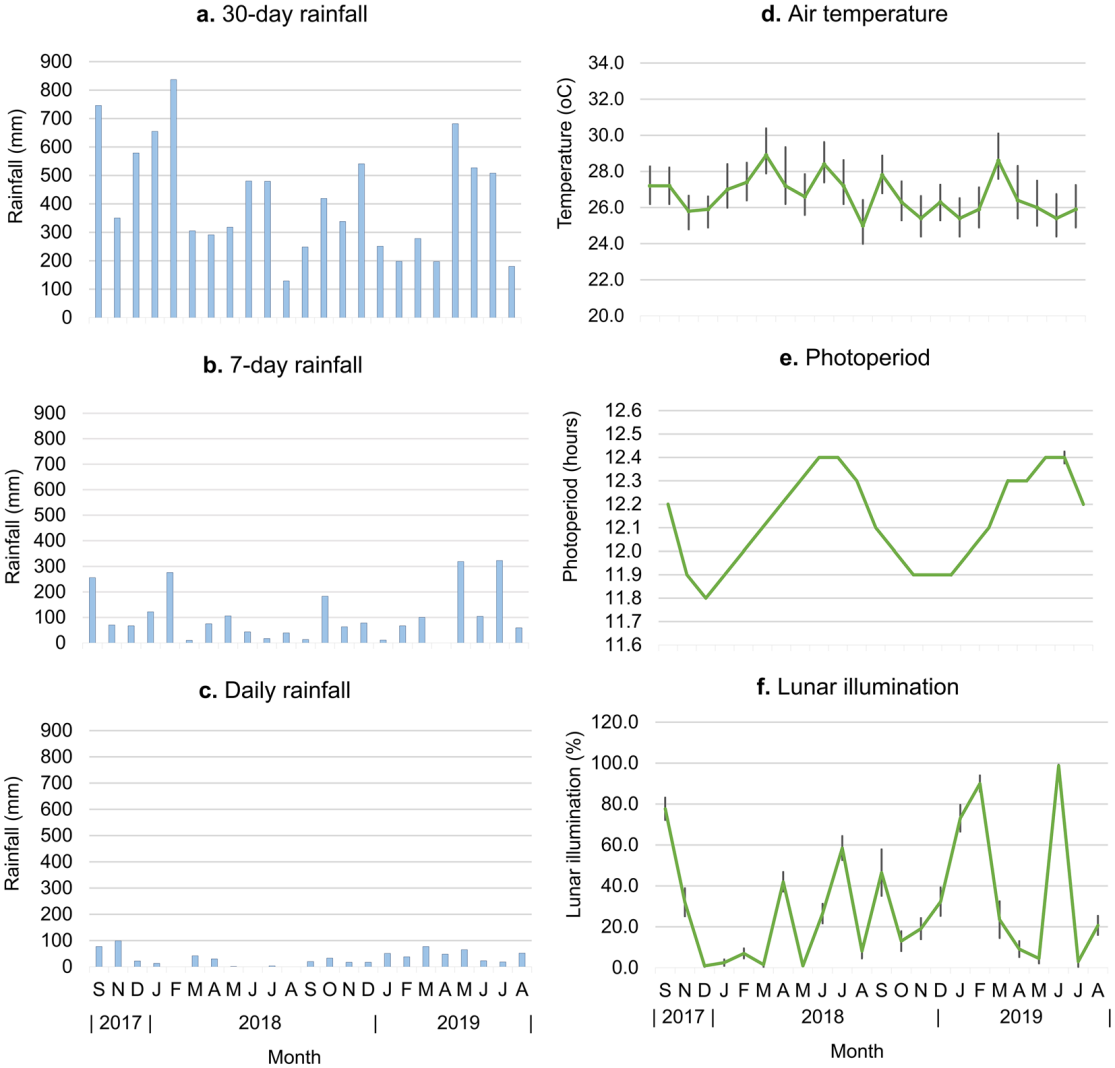
Monthly variations in environmental factors in: (**a**) 30-day rainfall, (**b**) 7-day rainfall, (**c**) daily rainfall, (**d**) air temperature (mean ± SE), (**e**) photoperiod (mean ± SE), (**f**) lunar illumination (mean ± SE) in Ulu Temburong National Park, Brunei from September 2017 to August 2018.

The 30-day rainfall pattern showed weak seasonal variations (Fig. 2a). The main period of high rainfall occurred in September 2017 and December 2017 to February 2018 (579–837 mm of rainfall). From March 2018, the 30-day rainfall varied between periods of low and moderate rainfall until August 2019. The 7-day rainfall and 30-day rainfall showed similar monthly variations (Fig. 2a,b). The daily rainfall did not correspond to the 30-day or the 7-day rainfall patterns (Fig. 2a–c), indicating variable daily rainfall distributions.

The mean air temperatures were constantly high throughout the study months with small fluctuation from 25 ± 1.14 °C to 28.9 ± 1.48 °C (Fig. 2d). Photoperiod showed a small annual range and varied monthly from 11.8 ± 0.0002 to 12.4 ± 0.0003 h (Fig. 2e). Increasing photoperiod occurred in January to July and decreasing photoperiod occurred in July to December. Lunar illumination showed a cyclical pattern of waxing (0–100% illumination) in and waning (100–0% illumination) phases of the lunar cycle (Fig. 2f). Eleven of the study months (January, February, April, July and September 2018 and November, December, January, February, June and August 2019) occurred during the waxing phases of the lunar cycle.

### Effect of environmental factors on spawning

Based on the GLM analyses of, spawning in *L. ovalis, R. argyrotaenia* and *T. tambra* were not found to be associated with any of the environmental factors (Table 2) that were studied. In all the three species, the odds of spawning male fishes were significantly higher than in females (OR = 9.960, p < 0.0001 in *L. ovalis*; OR = 10.489, p < 0.0001 in *R. argyrotaenia*; OR = 65.756, p < 0.0001 in *T. tambra*).

**Table 2:** Results of GLM of the effect of environmental variables on spawning in: (a) *L. ovalis*, (b) *R. argyrotaenia* and (c) *T. tambra*. Significant P-values are highlighted by*. OR: Odds ratio. OR = 1: Predictor variable does not affect the odds of response variable occurring. OR > 1: There is higher odds of spawning occurring in response to the predictor variables. OR < 1: There is lower odds of spawning occurring in response to the predictor variables.

Variable	Adjusted OR	95% interval OR	χ^2^ stat. (df)	P-value
(a) *L. ovalis*				
Sex (male)	9.960	(6.731, 14.812)	149.612 (1)	< 0.0001*
7-day rainfall	1.001	(0.999, 1.004)	1.637 (1)	0.072
Daily rainfall	0.999	(0.978, 1.019	0.012 (1)	0.913
Air temperature	0.78	(0.619, 1.012)	4.305 (1)	0.056
Lunar illumination	0.997	(0.988, 1.004)	0.720 (1)	0.396
Photoperiod	0.412	(0.167, 1.040)	3.465 (1)	0.064
(b) *R. argyrotaenia*				
Sex (male)	10.489	(6.302, 18.351)	107.122 (1)	< 0.0001*
7-day rainfall	0.989	(0.996, 1.001)	0.824 (1)	0.364
Daily rainfall	1.005	(0.993, 1.016)	0.610 (1)	0.435
Air temperature	0.845	(0.683, 1.042)	2.488 (1)	0.110
Lunar illumination	1.002	(0.995, 1.009)	0.320 (1)	0.572
Photoperiod	0.403	(0.141, 1.126)	3.003 (1)	0.083
(c) *T. tambra*				
Sex (male)	65.756	(16.181, 353.198)	4.194 (1)	< 0.0001*
7-day rainfall	1.000	(0.991, 1.009)	0.002(1)	0.960
Daily rainfall	0.900	(0.747, 1.007)	3.217 (1)	0.073
Air temperature	0.680	(0.294. 1.514)	0.902 (1)	0.342
Lunar illumination	1.001	(0.978, 1.025)	0.009 (1)	0.926
Photoperiod	1.011	(0.195, 1.682)	0.001 (1)	0.980

## Discussion

The present study is the first to describe the spawning periods of *L. ovalis*, *R. argyrotaenia* and *T. tambra*, which were found to be continuous throughout the year as revealed by the assessment of gonad maturity. Although spawning female *T. tambra* were only found in four months, the extended presence of females that had recently spawned and in preparation of spawning, as well as mature males supported the extended spawning strategy. Compared to the temperate region, considerably less research has examined the reproductive timing of freshwater fishes in tropical Asia. Temperate cypriniform fishes often display spawning seasonality due to the temporally variable temperature and photoperiod as reported in *Hybognathus placitus*^39^, *Barbus sclateri*, *Chondrostoma polylepis willkommii*^4^, *Cyprinus carpio*^4,6^ and *Notropis buccula*^40^. While, non-seasonal spawning reported in tropical cypriniform species such as *B. nigrofasciatus*, *B. dorsalis*, *B. titteya*^9^, *N. soroides*^18^ and *R. tawarensis*^19^ are often linked with temporally stable rainfall and temperature.

**Figure 3:**
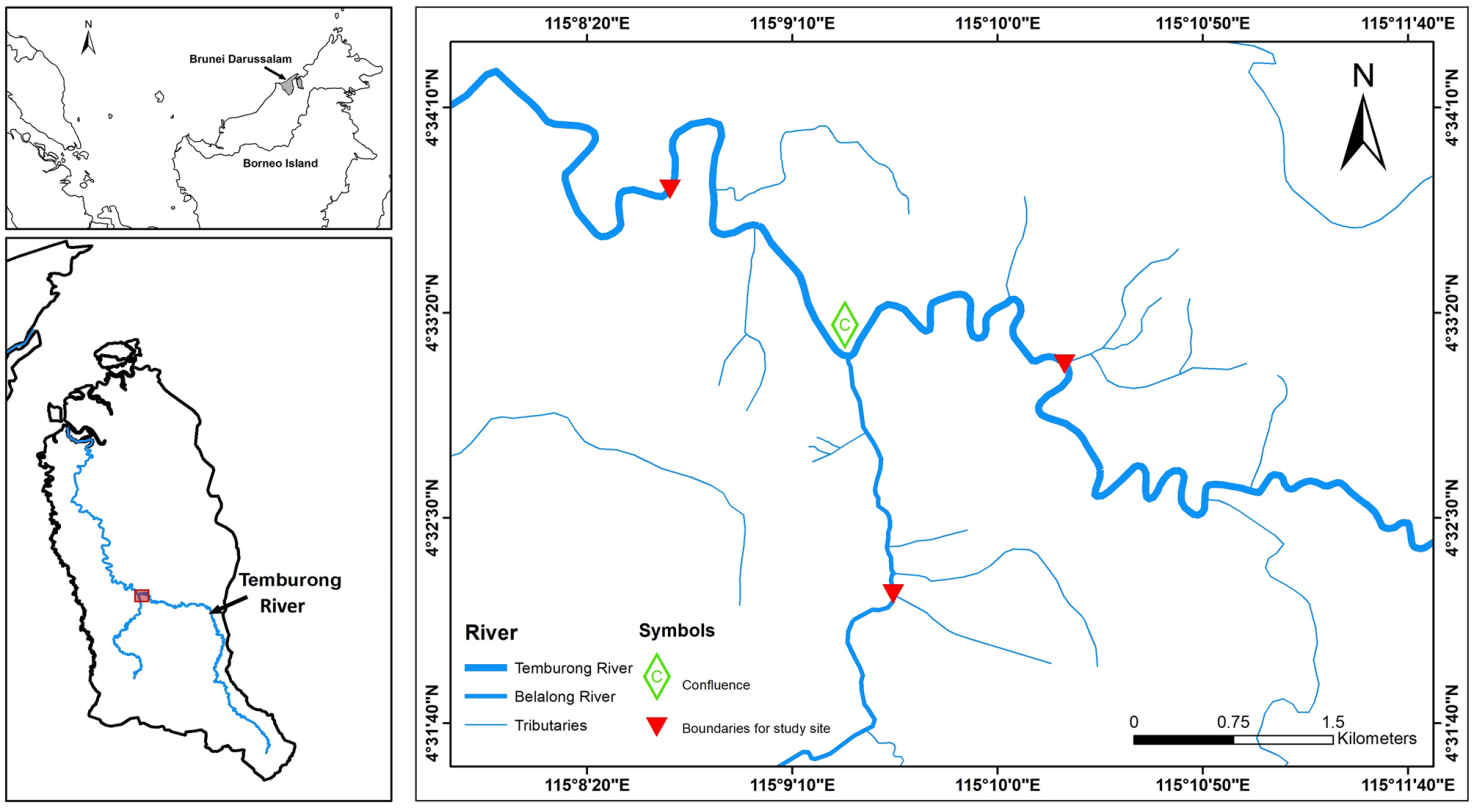
Study sites in the middle course of the Temburong River catchment in Brunei Darussalam, Northwest Borneo. Map created using ArcGIS Desktop Release 10 software with maps from free and open source QGIS.

In this study, the year-round spawning in *L. ovalis*, *R. argyrotaenia* and *T. tambra* also displayed highly variable spawning frequencies between months. Our analyses did not find that these spawning variabilities to be associated with any of the environmental factors that were investigated. Thus, the year-round spawning that was observed in these cypriniform fishes during this study could be related to the absence of distinct seasonal variations in rainfall, air temperature and photoperiod in the study site (Fig. 3).

Reproductive activities in tropical freshwater fishes have often been linked to rainfall pattern. Prolonged spawning has often been reported in freshwater fishes inhabiting regions with temporally stable rainfall such as in *B. nigrofasciatus*, *B. dorsalis*, *B. titteya* found in two river systems in Sri Lanka^9^. Other tropical fishes such as *N. soroide*s and *R. tawarensis* Malaysia and Indonesia respectively were reported to display peak spawning activities along with year-round spawning pattern. Although these regions do not have distinct wet and dry seasons, the spawning peaks were found to be linked with periods of high rainfall^18,19^. On the other hand, in tropical regions of South America with distinct seasonality in rainfall, protracted spawning in freshwater fishes such as *Gasteropelecus sternicla*, *Corynopoma riisei*, *Astyanax bimaculatus*, *Hemigrammus unilineatus*, *Corydoras aeneus*^41^; *Hypostomus pusarum*, *Cichla monoculus*, *Prochilodus brevis* and *Cichlasoma orientale*^42^ were reported to occur during the wet season.

Our results also showed how closely related species from distinct geographical locations may display different temporal spawning pattern. For example, *T. tambroides* which is a sympatric species of *T. tambra* in Peninsular Malaysia and Indonesia, displayed seasonal spawning in contrast to the continuous spawning that we reported for *T. tambra*. Ingram et al.^43^ reported a short spawning season of one to two months in *T. tambroides* in the Penisular Malaysia. Whereas Wibowo and Kaban^20^ reported protracted spawning season of six to eight months in *T. tambroides* in Indonesia. In both geographical locations, the spawning seasons in *T. tambroides* were linked to the wet season^20,43^.

Thus, our finding conforms to the current knowledge of the association between rainfall and spawning in tropical freshwater fishes. In this study, we report that there was no distinct wet and dry season and rainfall was variable on a monthly, weekly and daily scales (Fig. 2). Heavy downpours can occur at any time of the year and due to the location of the study site in the upper catchment of the Temburong basin (Fig. 3), spates in response to heavy rains occur frequently^44^. The lack of seasonality in spawning in *L. ovalis*, *R. argyrotaenia* and *T. tambra* could be attributed to the flashy and unstable hydrology of the Temburong basin. Magoulick et al.^45^ and Hitt et al.^46^ reported the association of unstable hydrology with the abundance of opportunistic strategists which comprised fishes that have prolonged breeding season and continuous or multiple breeding events^46,47^. Fishes that are opportunistic strategists can recolonise freshwater habitats efficiently following disturbances caused by hydrologic spates^2,48^. Thus, the non-seasonal reproduction observed in *L. ovalis*, *R. argyrotaenia* and *T. tambra* was possibly an evolutionary adaptation to ensure their survival in an unstable environment.

Air temperature is recognized as an important environmental cue for spawning in freshwater fishes at higher latitudes which show seasonal air and water temperature variations^5,7,49,50^. The role of air temperature in reproduction in tropical freshwater fishes is not well understood. On the contrary, water temperature has been shown to influence the temporal pattern of reproduction of freshwater fishes in other tropical regions. For instance, in a semi-arid region in Northeast Brazil where water temperature showed variations of 24.5–31 °C, peak spawning activity in *Cichla monoculus* was found to coincide with lower water temperature^23^. While, in the tropical wetlands of West Bengal India water temperature range of 29–31 °C was found to be the most favorable for spawning in *Channa punctata*^12^. This study only looked at the effect of air temperature on spawning in the three studied species. We did not find significant associations of air temperature with spawning possibly because air temperature was constantly high throughout the study period which provided favorable conditions for continuous spawning in *L. ovalis*, *R. argyrotaenia* and *T. tambra*^51^. The effect of water temperature on spawning was not investigated in this study and should be a subject of further investigation.

The role of photoperiod as important environmental cue for spawning in fishes is well-known at higher latitudes^5,7,49,50^. Although less frequently reported, photoperiod have been shown to trigger spawning migrations in neotropical riverine fishes^24,25^. For example, spawning migration in *Prochilodus argenteus* was reported to coincide with longer photoperiods^10^. Variations in photoperiod influence gonad maturation and affect spawning frequencies as seen in *Catla catla*, *Betta splendens*^52^ and *Oreochromis niloticus*^53^. However, in this study we did not find evidence of spawning being association with photoperiod which could be attributed to the insignificant temporal differences in photoperiod.

Our result also showed that sex is an important predictor of spawning where males fishes were much more likely to spawn throughout the year in comparison to the females. The continuous presence of the spawning capable male is likely a reproductive strategy which ensures that males were physiologically ready to spawn when the first mature females became available. Similar year-round presence of reproductively active male fishes has been reported in other freshwater fishes such as in live-bearing poecilid fishes in Costa Rica^54^. This reproductive strategy is thought to be facilitated by the reduced energetic cost of producing spermatozoa compared to oocytes in females^14^.

This study presents new information on reproductive ecology of *L. ovalis* and *R. argyrotaenia* and *T. tambra* and adds to the knowledge on freshwater fishes in Southeast Asia and the tropical region. The spawning periods of these cypriniform species were found to extend throughout the year and were linked to the temporally stable rainfall, air temperature and photoperiod thus providing further evidence for non-seasonal reproduction in tropical Asian freshwater fishes. Our finding also provided clues on the life history strategies of tropical cypriniform fishes which inhabit flashy and unstable headwater streams and thus suggesting the utility life history knowledge for understanding ecological responses in freshwater fishes in the context of habitat and climate changes. In consideration of the small sample size of male *T. tambra*, we recommended that further studies are needed to elucidate whether male *T. tambra* are reproductively active throughout the year. Further, we recommend that water temperature is also examined as one of the environmental factors related to spawning. In addition, experimental studies related to temperature effects on gonadal maturation will aid in better understanding the mechanism of reproductive activities in tropical freshwater fishes.

## Materials and methods

### Site description

The study site was located in the upper catchment of the Temburong River Basin (1100 km^2^) in Brunei Darussalam (Brunei) in Northwest Borneo (4° 33′ 8.77″ N, 115° 9′ 24.21″ E; Fig. 3), within Ulu Temburong National Park (UTNP), a protected area that is dominated by the Mixed Dipterocarp Forest. Brunei has a tropical climate, which is weakly influenced by the Southeast Asian monsoon. UTNP receives at least 4000 mm of rainfall annually with weakly seasonal rainfall consisting of two maxima (October to January and May to July) and two minima (February to March and June to August)^44,55^. However, daily weather in Ulu Temburong is highly variable as a result of localized storms. The rivers are characterized by swift and turbulent water current with coarse substrates, as well as the prevalence of rapids and waterfalls^44^ The study site comprised the primary channels of the Temburong and Belalong Rivers that were 20–50 m wide, and where flow was perennial. Sampling stations were chosen based on accessibility and were found within 3 km from the confluence of the two rivers and were 40–80 m above sea level.

### Sample collection

Sampling for *L. ovalis*, *R. argyrotaenia* were conducted monthly for a period of 24 months from September 2017 to August 2019, with the exception of October 2017. While, *T. tambra* was sampled for 15 months from July 2018 to August 2019. Multi-meshed gill nets were used for sampling *L. ovalis* and *R. argyrotaenia* which were constructed by combining gill nets of two different mesh sizes e.g., 2.5 and 3.8 cm inch mesh sizes. The multi-meshed gill nets were 1.5 m deep and 3.5 m wide and mesh sizes 2.5, 3.2, 3.8 and 4.4 cm were used for *L. ovalis*, and 0.6 and 1.9 cm for *R. argyrotaenia*. For *T. tambra,* gill nets were 2.5 m deep, 6.5 m wide and consisted of 6.4, 7.6 and 8.9 cm mesh sizes.

The sampling duration in each month takes about three to five days to collect up to 30 specimens of each fish species. For *L. ovalis* and *R. argyrotaenia*, sampling was conducted during day time and the multi-meshed gill nets were deployed in suitable habitats for up to 8 h per day. While for *T. tambra* sampling was conducted in daylight or overnight where gill nets were deployed in suitable habitats for up to 14 h per day. All captured fishes were recorded and counted and those not sampled were released back to the river. Collected specimens of *L. ovalis*, *R. argyrotaenia* and *T. tambra* were euthanized using MS-222 (Tricaine methanesulfonate), stored on ice and taken to the laboratory for further procedures.

### Morphological measurements and GSI

All fishes were measured for total length, body weight and gonad weight. Total length (TL) is the length of the fish from the tip of the snout to the end of the tail and was measured using a scale to the nearest mm. Body weight was measured to the nearest 0.1 g. Whole gonads were carefully removed from the visceral cavity and weighed the nearest 0.01 g. Weight measurement were made using a top pan balance (Nimbus, NBL 2602e). The mean monthly TL ± standard error (SE) was calculated for the females and males of each species. Mean monthly GSI ± SE were calculated for the females and males of each species. GSI is a measure of gonad maturity, where high GSI values indicate advanced of gonad maturation and vice-versa, which was calculated as follows for each fish:

$${\text{GSI}} = \frac{{{\text{Gonad}}\;{\text{weight}}({\text{g}})}}{{{\text{Total}}\;{\text{body}}\;{\text{weight}}({\text{g}})}} \times 100$$

### Gonad histology and reproductive phases

Histological preparations of the gonad tissues followed traditional paraffin wax embedding techniques. A transverse section about 1 cm thick, were taken from the anterior, medial and posterior portion of one gonad of each fish. These sections were placed in 10% neutral buffered formalin for about 2 weeks to allow sufficient tissue fixation. Fixed tissues were dehydrated in a series of ethanol dilutions cleared in xylene and embedded in liquid paraffin wax. The embedded samples were sectioned with a microtome to obtain 4–6 μm-thick sections which were mounted on glass slides. The paraffin sections were dewaxed in xylene, stained with Harris haematoxylin and eosin-Y and then cover-slipped with DPX as the mounting medium^56^. All slides were observed using a microscope (Leica DM 750) to determine the sex and reproductive phases of the gonad.

Gonad development was assessed by classifying ovaries and testes of each species into reproductive phases following Brown-Peterson et al.^57^ with a slight modification. Six instead of five phases were identified in this study, namely: (1) Immature, (2) developing, (3) spawning capable, (4) actively spawning, (5) regressing, and (6) regenerating. According to Brown-Peterson et al.^57^ the ‘spawning capable’ phase includes fishes that are physiologically able to spawn within the current reproductive cycle due to advanced gamete development. The spawning capable phase also includes the ‘actively spawning’ subphase, in which a gonad is in the most advanced stage of gamete maturation where spawning is imminent or taking place. In this study, the ‘actively spawning’ was assigned as a main phase in addition to the ‘spawning capable’ phase to distinguish fishes that were spawning from those that were sexually mature but were not spawning, making six phases instead of five. The actively spawning phase was used as an indicator of spawning.

Each gonad was assigned a reproductive phase following the classification above. Monthly frequencies (expressed as percentage) of the maturity levels were calculated for the females and males of each species, as follows:

$${\text{Maturity}}\;{\text{level}}\;{\text{frequency}}\;(\% ) = \frac{{{\text{N}}_{{\text{p}}} }}{{{\text{N}}_{{\text{t}}} }} \times 100$$

where Np was the number of fishes in a particular phase of a particular month, and N_t_ was the total number of fishes in a particular month.

### Environmental factors

Environmental factors of interest included rainfall, air temperature, photoperiod and lunar illumination which were recorded on the sampling days of each month. Rainfall and air temperature data from September 2017 to August 2019 were obtained from a weather station (WeatherHawk 240 Signature Wireless Weather Station) at the Kuala Belalong Field Studies Centre which was located within the study site. Data on photoperiod and lunar illumination for Brunei in 2017–2019 were obtained from a website^58^. Photoperiod was the numbers of daylight hours from sunrise to sunset on each day. While lunar illumination represented the percentage (0–100%) of the moon's disk that appeared illuminated on each day.

Rainfall was expressed as the total amount of rain in millimetres (mm) within a specified period. To capture the variations in rainfall along different temporal scales 30-day rainfall, 7-day rainfall and daily rainfall patterns were examined. The 30-day and 7-day rainfall were calculated by summing the rainfall on the first sampling day of a particular month and the preceding 29 and 6 days, respectively. The daily rainfall was calculated by summing the rainfall on sampling days in a particular month.

The values of each parameter on each sampling day values were used in the GLM analyses. For monthly values of environmental factors, the total values of the three rainfall variables were calculated for each sampling month. While, for air temperature (°C), photoperiod (hours) and lunar illumination (%) monthly mean ± SE were calculated.

### Data analyses

All statistical analyses were performed using R Statistical Software, version 4.1.3^59^.

#### Sex composition

Overall sex compositions were subjected to the Chi-Square test at α = 0.05 to detect significant deviations from the expected 1:1 female to male sex ratio.

#### TL, GSI and reproductive phases

Differences in the TL and GSI between the male and female of each species and the differences in the GSI between reproductive phases were explored through linear mixed-effects (LME) modelling and the nlme package, version 3.1-155^60^. Three separate models were explored to assess the effect of sex on: (a) TL of *L. ovalis*, (b) TL of *R. argyrotaenia* and (c) TL of *T. tambra*. Another three separate models were explored to assess the effect of sex and reproductive phases on: (a) GSI of *L. ovalis*, (b) GSI of *R. argyrotaenia* and (c) GSI of *T. tambra*. For all models, the sampling month was modelled as the random effect. Where necessary, the response variable was log 10-transformed to meet the assumption of normal distribution prior to analysis. Following each of the LME analysis, a pair-wise comparison was performed by obtaining the estimated marginal means (a.k.a. least squares means) using the emmeans package, version 1.7.1-1^61^. The pairwise comparison indicated the differences in means between the different groups that were being tested. We performed data exploration before each LME model followed with model validation after executing the LME model according to the protocol by Zuur et al.^62^. Diagnostic plots of the final LME models in Supplementary Figs. S2–S7 are available online.

#### Association of environmental factors with spawning

The association of environmental factors with spawning activities were explored through a generalised linear model (GLM) using the binomial family function. This model was used to predict the binary outcome of spawning i.e., the odds of spawning occurring in response to the environmental factors. Three models were explored to examine the effect of environmental factors on spawning in: (a) *L. ovalis*, (b) *R. argyrotaenia* and (c) *T. tambra*. To fit the initial GLM models for each species, the actively spawning phase was modelled as the response variable while sex, 30-day rainfall, 7-day rainfall, daily rainfall, air temperature, photoperiod and lunar illumination were modelled as the predictor variables. The initial model was then checked for multicollinearity and where predictor variables with variable inflation factor (VIF) greater than 3 were removed. Subsequently, the final model was fitted with sex, 7-day rainfall, daily rainfall, air temperature, photoperiod and lunar illumination as the predictor variables and actively spawning phase as the outcome variable. We performed data exploration before each GLM followed with model validation after executing the GLM according to the protocol by Zuur et al.^62^ Diagnostic plots of the final GLMs in Supplementary Figs. S8–S10 are available online.

### Ethics approval

Fish sampling and handling procedures were approved by the University Research Ethics Committee of Universiti Brunei Darussalam (approval reference: UBD/OAVCR/UREC/Feb18-09; 1 March 2018). All animal procedures were performed in accordance with the Universiti Brunei Darussalam guidelines on the use of animals for scientific procedures. All methods were performed in accordance with the ARRIVE guidelines.

## Supplementary Information

Supplementary Figures.

## Data Availability

The datasets analysed during the current study are available from the corresponding author upon reasonable request.
