# Supplementary material for: Year-round spawning of three tropical Cypriniformes fishes in Southeast Asia
Source: Sci Rep. 2023 Jun 2;13:8971. doi: 10.1038/s41598-023-36065-9 (PMC10238536; doi:10.1038/s41598-023-36065-9)
Supplement: Supplementary file 1 — Supplementary Figures. [file 41598_2023_36065_MOESM1_ESM.pdf]

SUPPLEMENTARY INFORMATION

Year-round spawning in Southeast Asian Cypriniformes fishes, *Lobocheilos ovalis*, *Rasbora argyrotaenia* and *Tor Tambra*

Rafhiah Kahar [1,*], Norhayati Ahmad [1,2] and Takaomi Arai [2*]

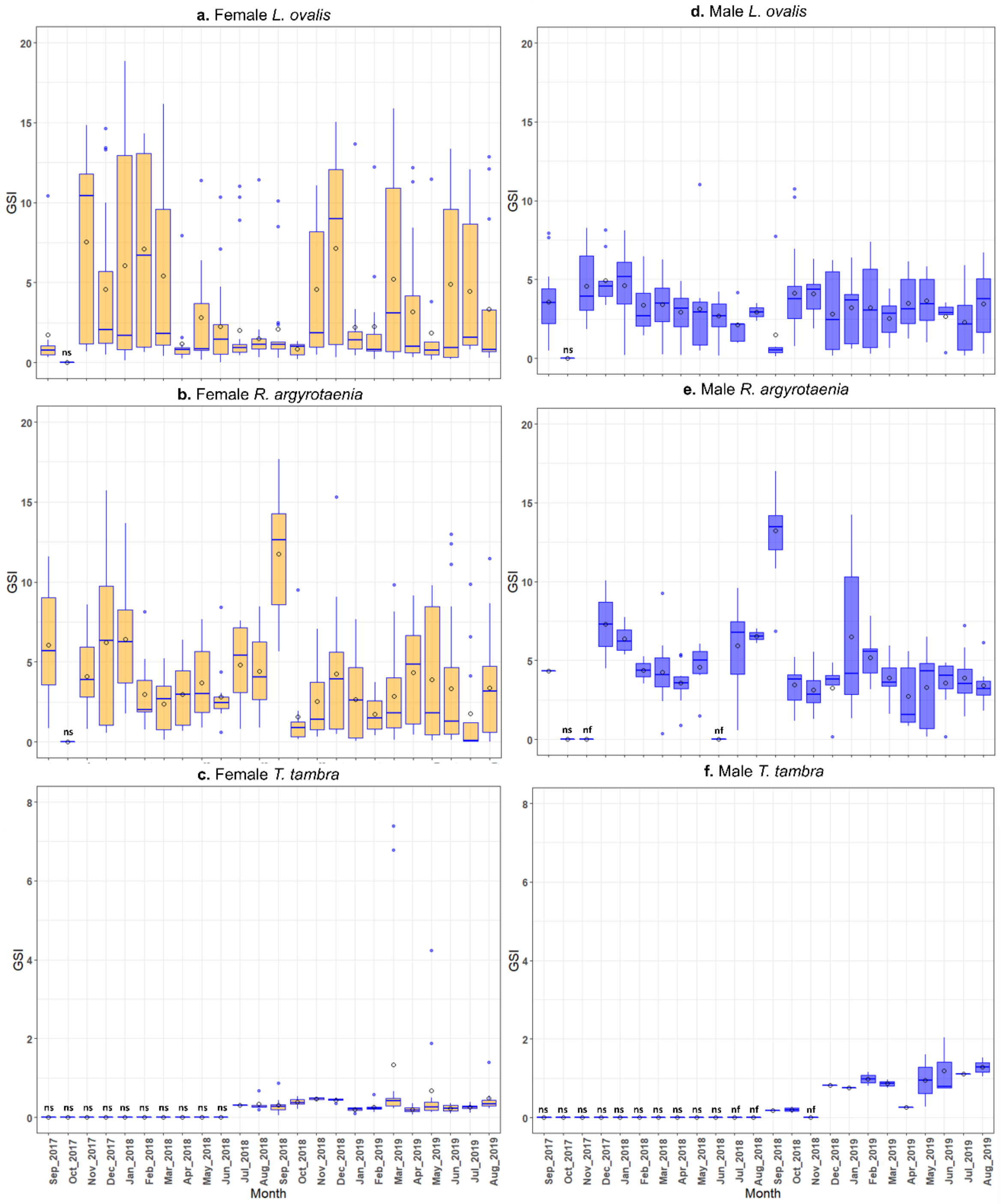

Supplementary Figure S1: Monthly fluctuations in gonadosomatic index (GSI) in: (a) Female *L. ovalis*, (b) female *R. argyrotaenia*, (c) female *T. tambra*, (d) male *L. ovalis*, (e) male *R. argyrotaenia*, (f) male *T. tambra* from September 2017 to August 2019. Hollow circle: mean GSI. ns: no study was conducted in the respective month. nf: study was conducted but no fish specimens were collected.

SUPPLEMENTARY INFORMATION

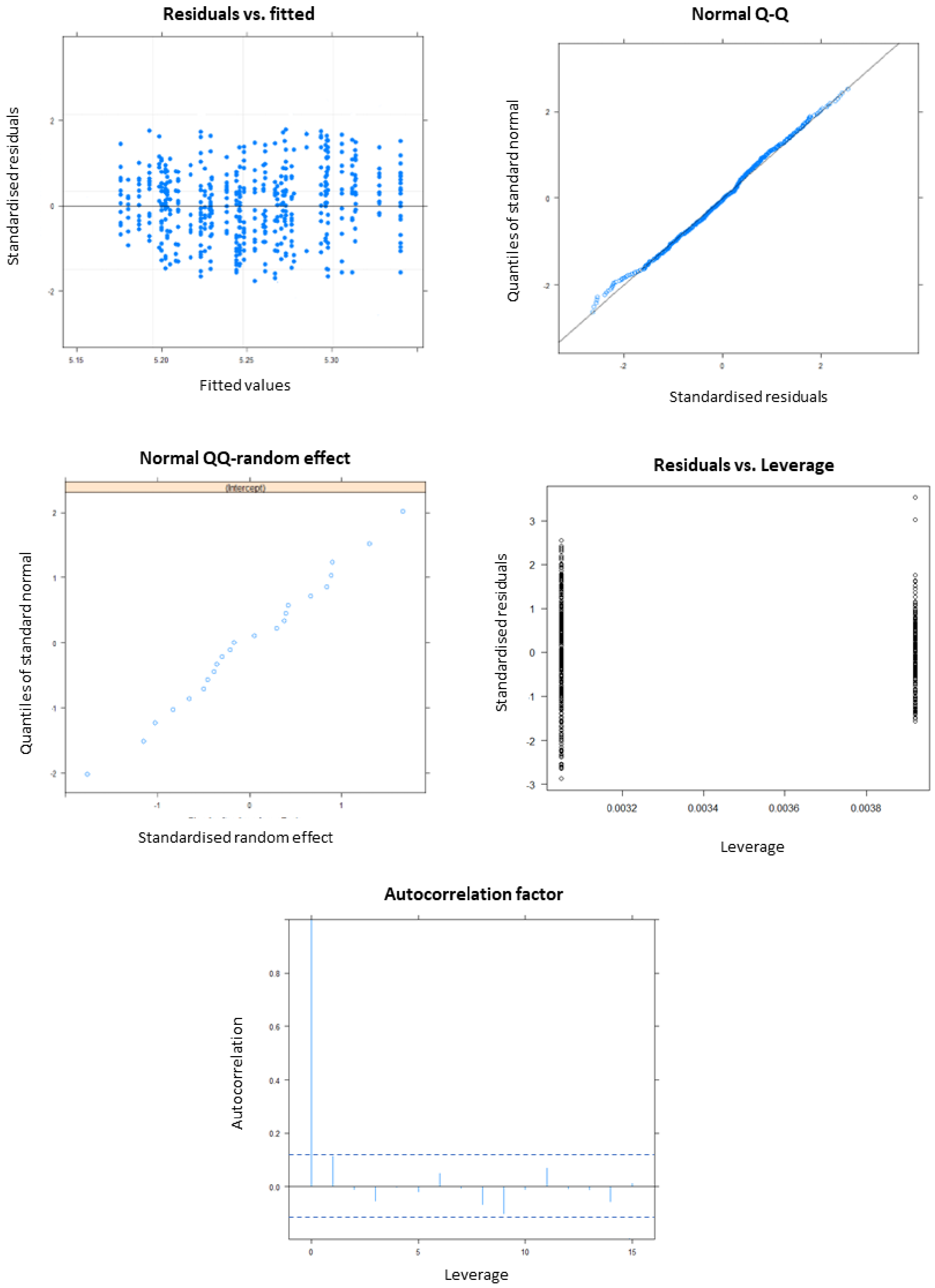

Supplementary Figure S2: Diagnostics plots for LME model of the effect sex on TL in *L. ovalis* showing the assumptions for the LME model are met, leading to a better fit of the model.

SUPPLEMENTARY INFORMATION

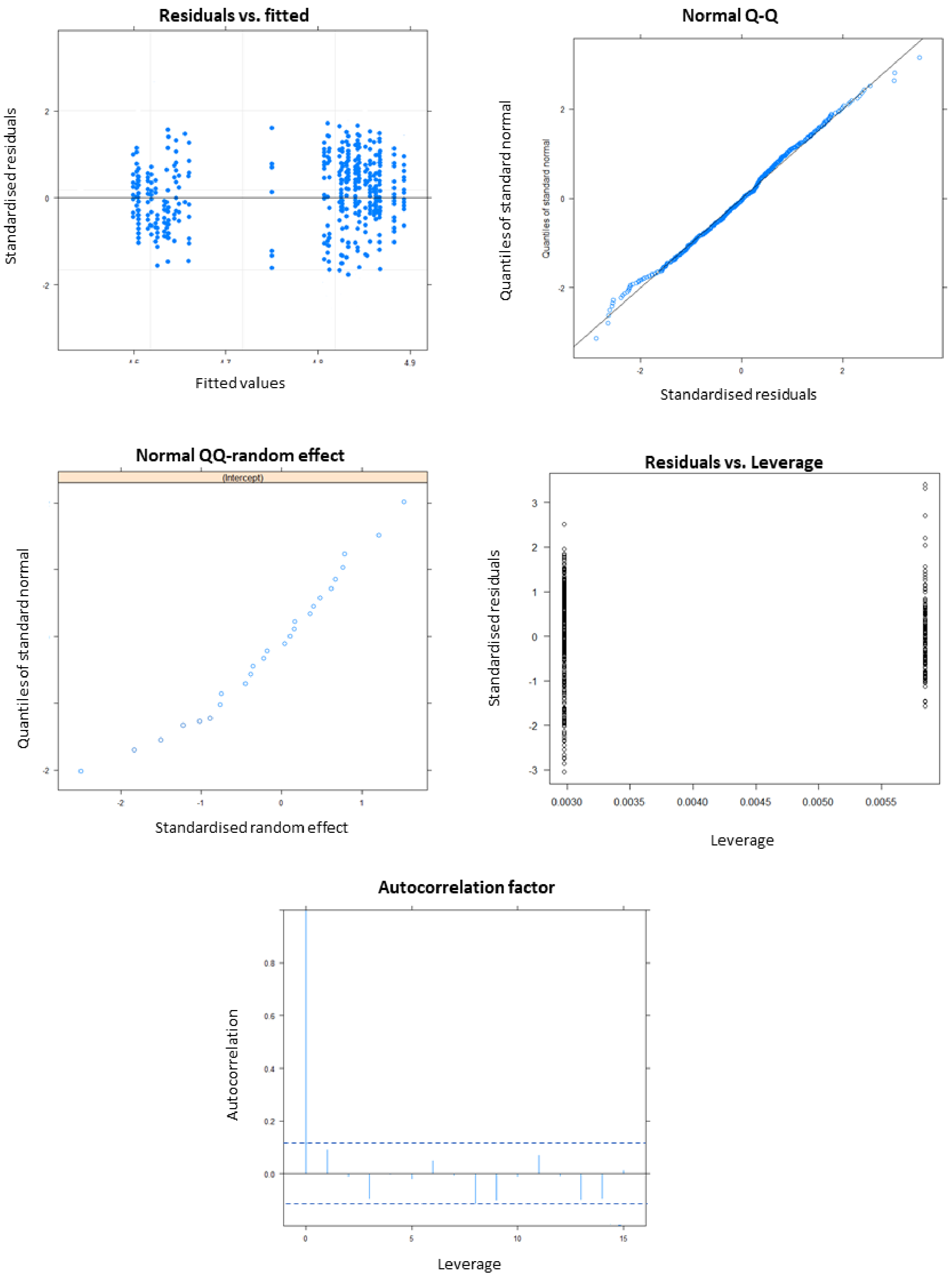

Supplementary Figure S3: Diagnostics plots for LME model of the effect sex on TL in *R. argyrotaenia* showing the assumptions for the LME model are met, leading to a better fit of the model.

SUPPLEMENTARY INFORMATION

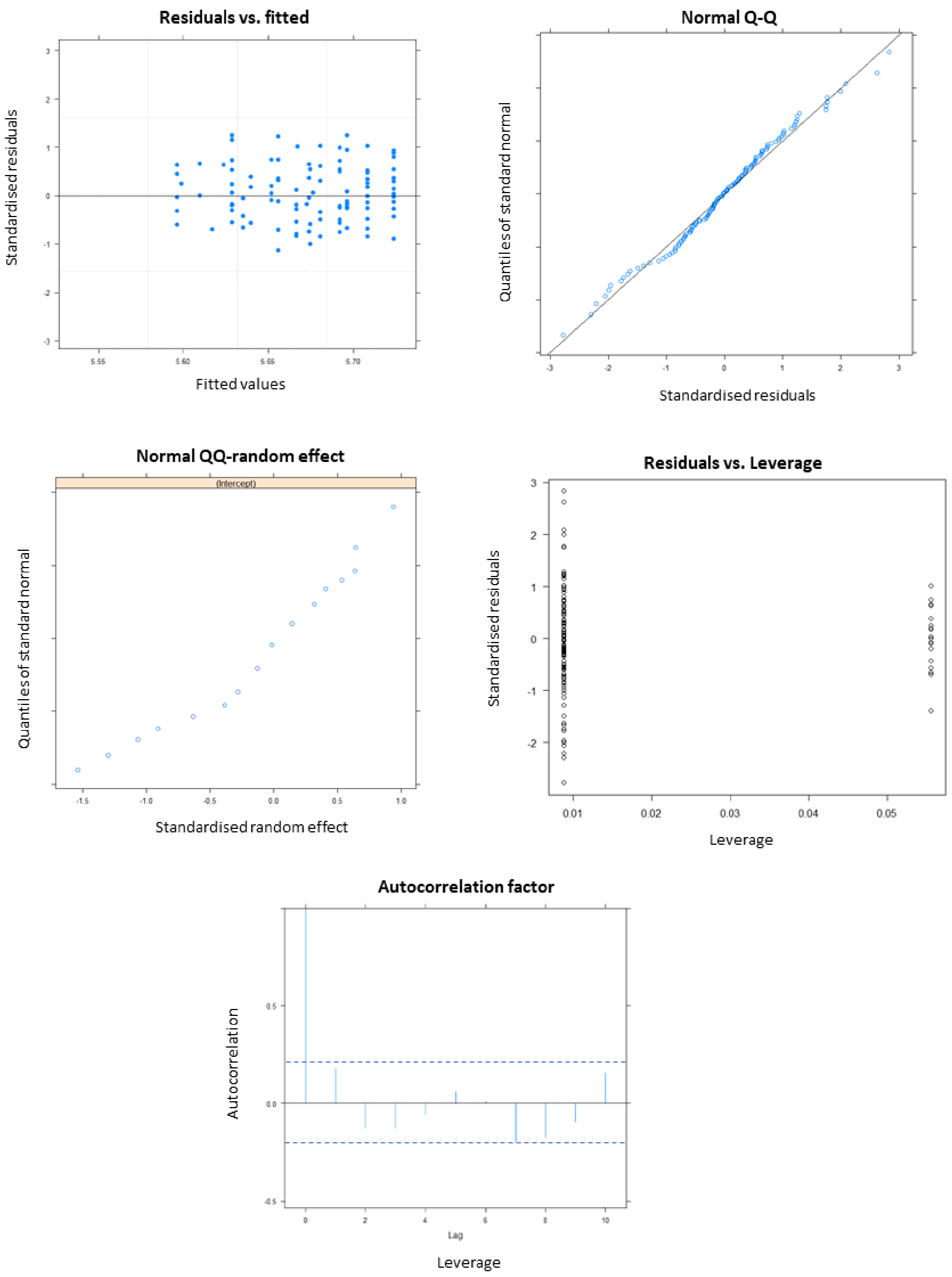

Supplementary Figure S4: Diagnostics plots for LME model of the effect sex on TL in *T. tambra* showing the assumptions for the LME model are met, leading to a better fit of the model.

SUPPLEMENTARY INFORMATION

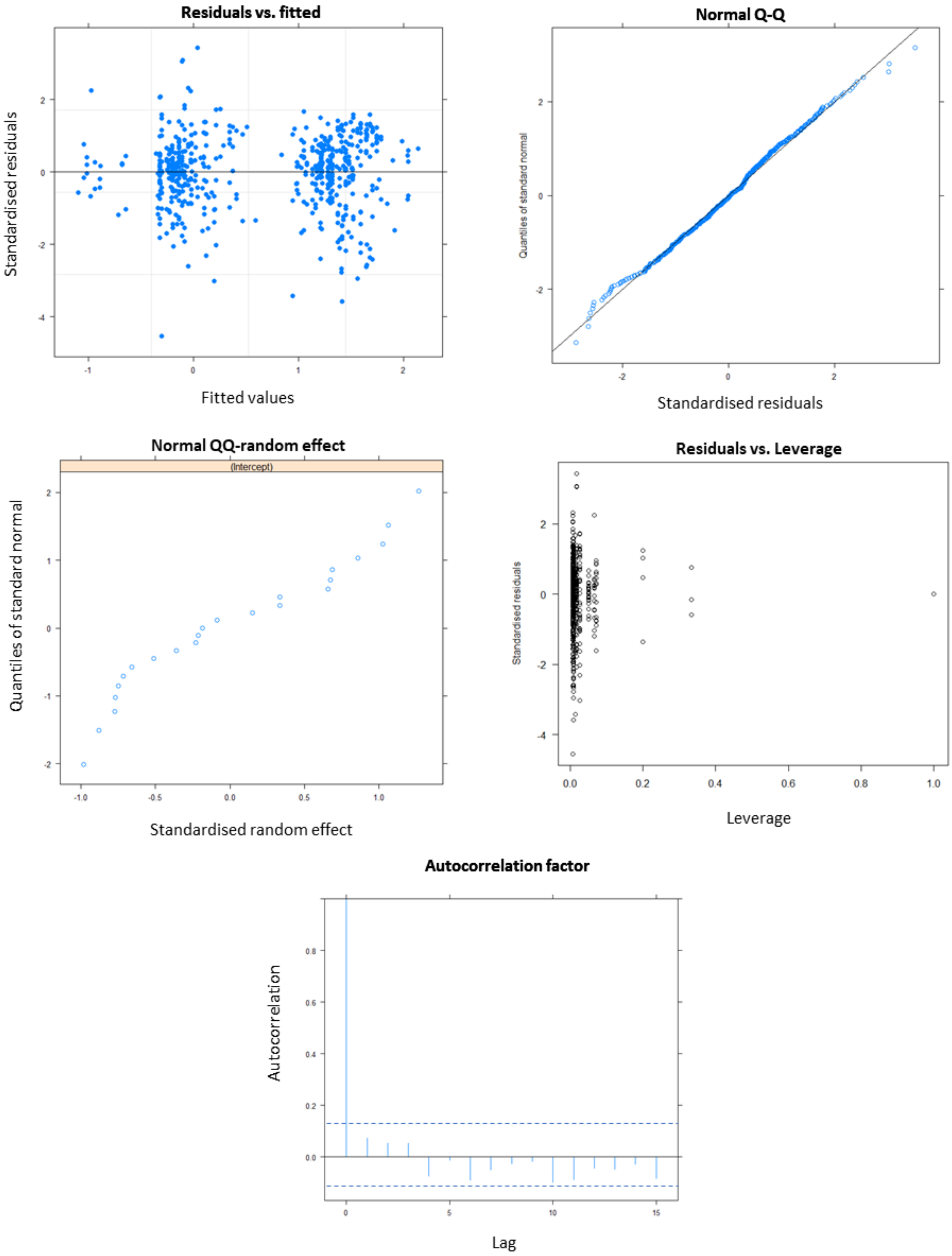

Supplementary Figure S5: Diagnostics plots for LME model of the effect sex and reproductive phase (maturity level) on GSI in *L. ovalis* showing the assumptions for the LME model are met, leading to a better fit of the model.

SUPPLEMENTARY INFORMATION

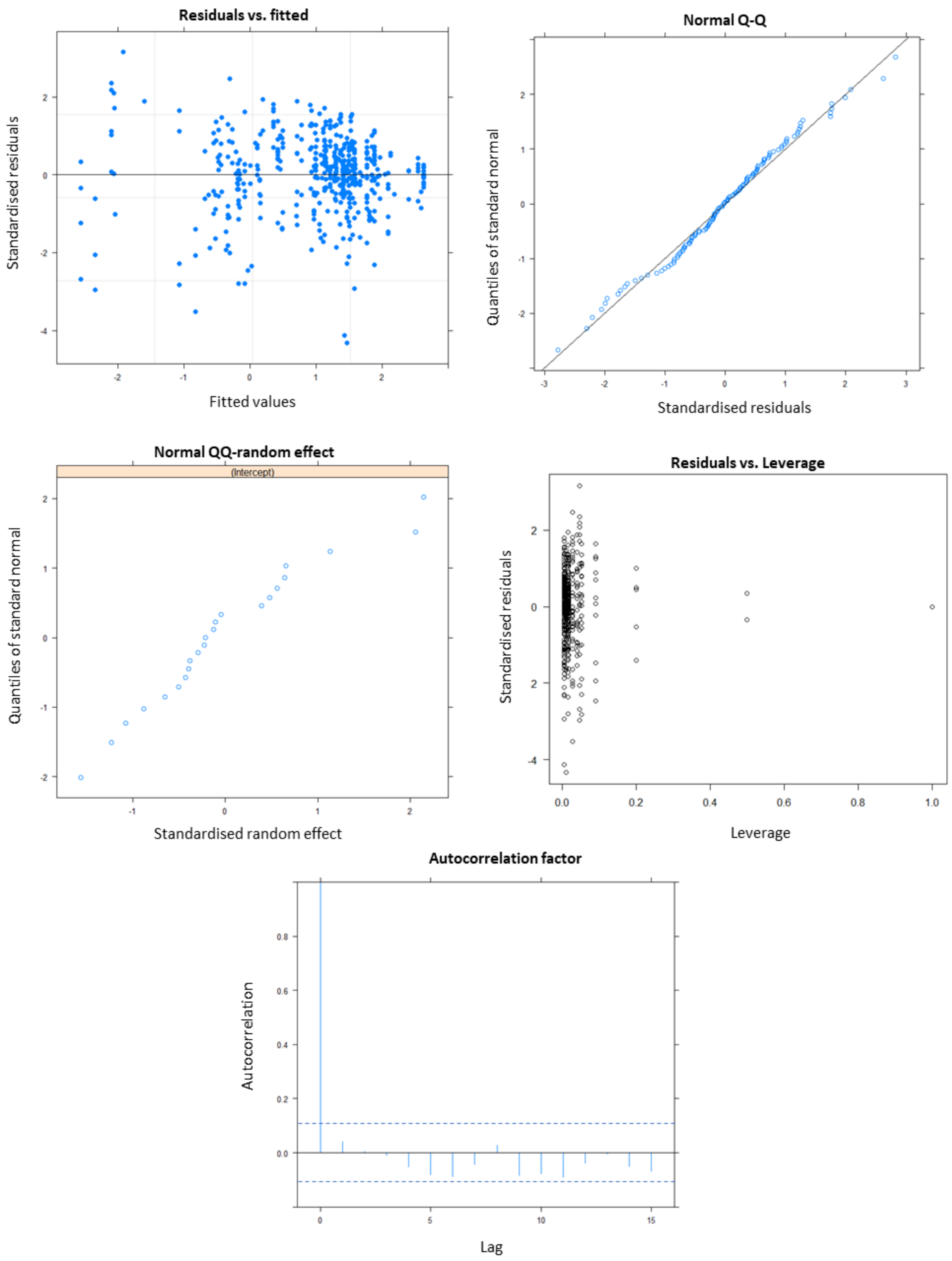

Supplementary Figure S6: Diagnostics plots for LME model of the effect sex and reproductive phase (maturity level) on GSI in *R. argyrotaenia* showing the assumptions for the LME model are met, leading to a better fit of the model.

SUPPLEMENTARY INFORMATION

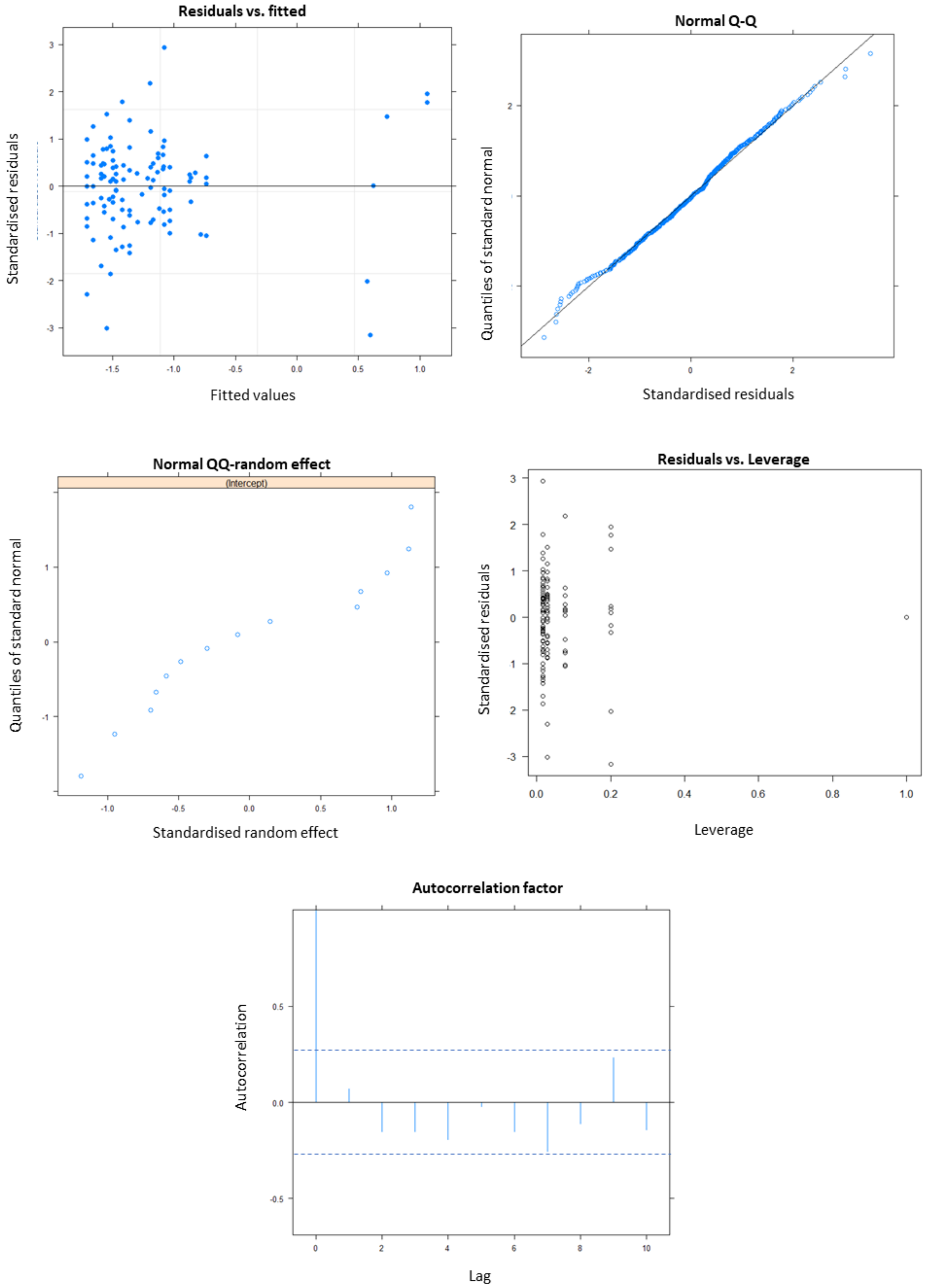

Supplementary Figure S7: Diagnostics plots for LME model of the effect sex and reproductive phase (maturity level) on GSI in *T. tambra* showing the assumptions for the LME model are met, leading to a better fit of the model.

SUPPLEMENTARY INFORMATION

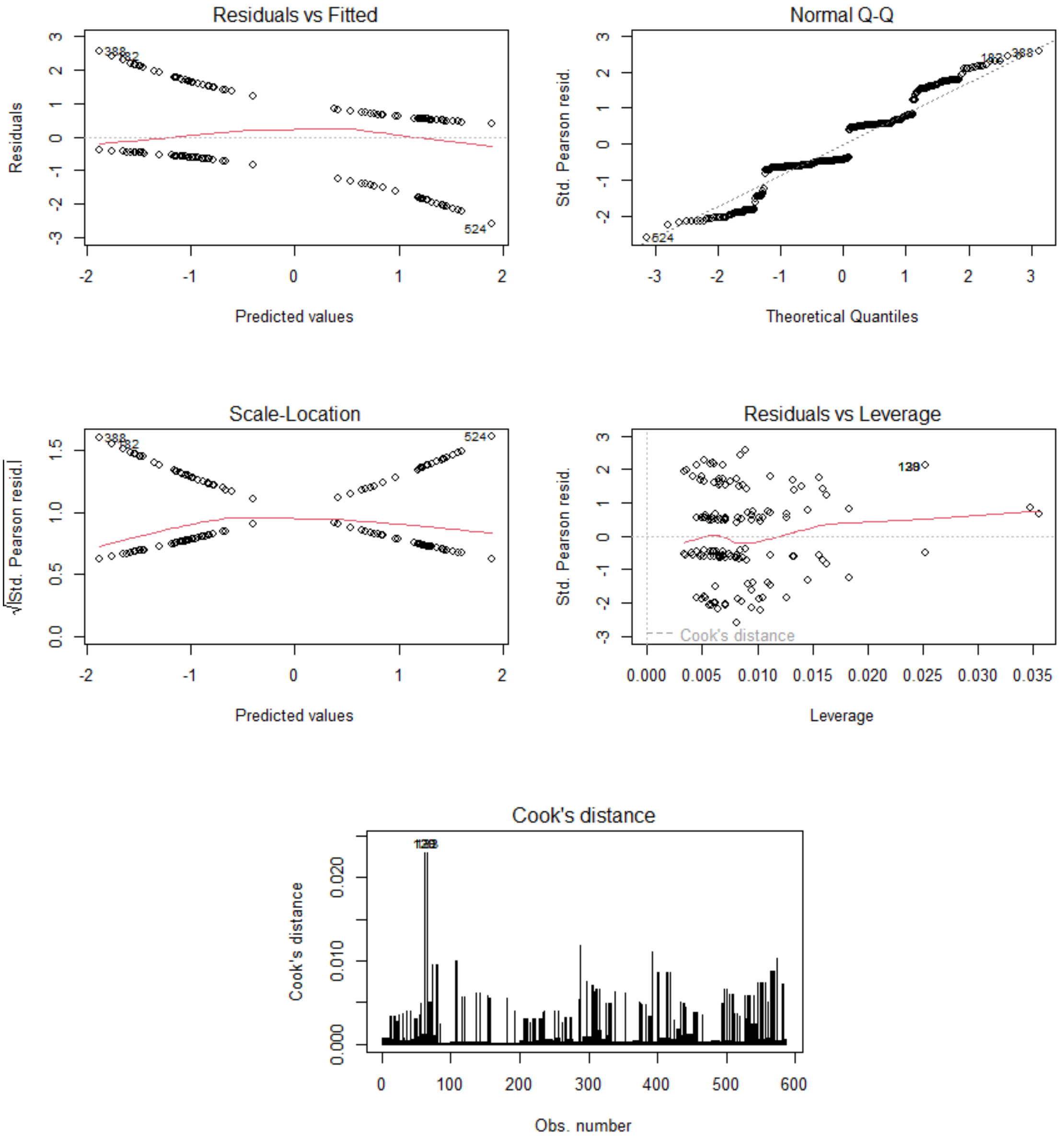

Supplementary Figure S8: Diagnostics plots for the GLM of the effect of environmental factors on spawning in *L. ovalis*, showing the assumptions for the logistic regression are met, leading to a better fit of the model.

SUPPLEMENTARY INFORMATION

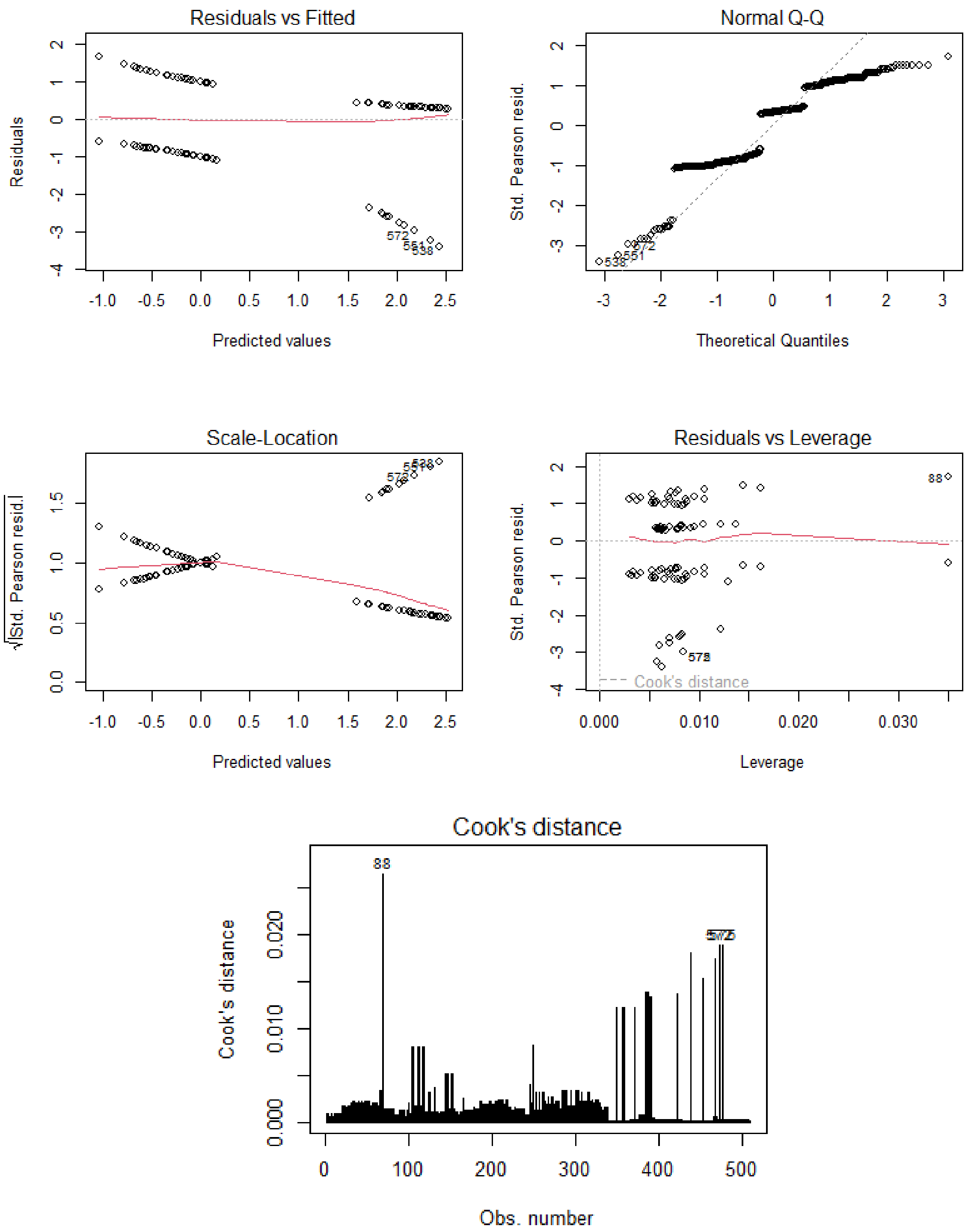

Supplementary Figure S9: Diagnostics plots for GLM of the effect of environmental factors on spawning in *R. argyrotaenia*, showing the assumptions for the logistic regression are met, leading to a better fit of the model.

SUPPLEMENTARY INFORMATION

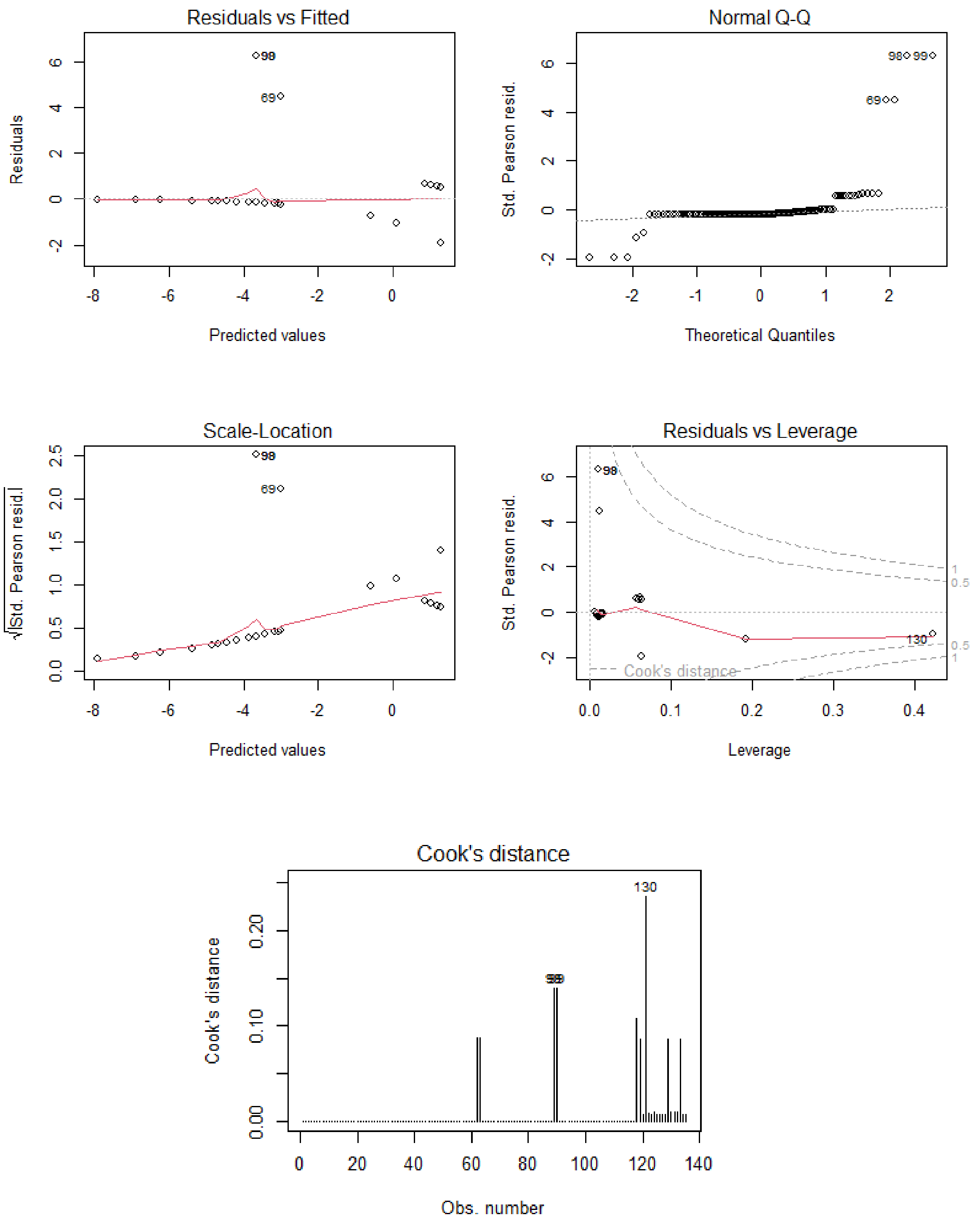

Supplementary Figure S10: Diagnostics plots for GLM of the effect of environmental factors on spawning in *T. tambra*, showing the assumptions for the logistic regression are met, leading to a better fit of the model.
